# The inhibiting impact of artificial facility intrusion on tourists’ pro-environmental behavior

**DOI:** 10.1038/s41598-026-52902-z

**Published:** 2026-05-14

**Authors:** Yinzhong Gou, Yue Liu, Tingting Wang, Xingguang Pan, Yijun Liu

**Affiliations:** 1 College of Business and Economics, Australian National University, Canberra, 0200 Australia; 2 Business School, Sichuan University, Chengdu, 610065 China; 3 School of Business Administration, Faculty of Business Administration, Southwestern University of Finance and Economics, Chengdu, 611130 China; 4 Faculty of International Tourism Management, City University of Macau, Taipa, 999078 Macau China

**Keywords:** Artificial facility intrusion, Pro-environmental behavior, Aesthetic experience, Place attachment, Ecological environment value, Broken window effect, Environmental social sciences, Environmental studies, Psychology, Psychology

## Abstract

In the development and management of natural tourism destinations, the introduction of artificial facilities provides convenience. However, due to visual incongruity, these facilities may invade the aesthetic harmony of the natural landscape and, through the “broken windows effect,” inhibit tourists’ pro-environmental behaviors. Nevertheless, the internal psychological mechanism and boundary conditions of this phenomenon remain unclear. This study systematically explored the impact path of artificial facility intrusion on tourists’ pro-environmental behaviors through three progressive situational experiments. Study one confirmed the direct negative impact of artificial facility intrusion on pro-environmental behaviors. Study two revealed the chain mediating role of “aesthetic experience–place attachment” in this impact. Study three further verified the positive moderating role of individual ecological environment value in the “aesthetic experience–place attachment” path. The results show that artificial facility intrusion weakens tourists’ aesthetic experience and place attachment, thereby inhibiting their pro-environmental behaviors, while high levels of ecological environment value can significantly strengthen the positive transformation of aesthetic experience to place attachment. This study not only provides empirical evidence for understanding the behavioral transmission mechanism of “visual breakage” in the tourism environment but also provides important insights for optimizing facility planning in natural destinations.

## Introduction

During the tourism development and operation of natural scenic areas, supporting artificial facilities are often constructed to meet service demands and enhance tourists’ experiences^1^. Although such artificial facilities bring convenience to tourists, they have altered the natural landscape of scenic areas and constitute an intrusion into the natural environment. The intrusion of artificial facilities may visually disrupt the integrity and continuity of the landscape, weaken the inherent harmony and aesthetic appeal, and further trigger a series of negative consequences^2,3^. For instance, the Bailong Elevator in the Wulingyuan of Zhangjiajie, China, has elicited negative evaluations from tourists due to its direct integration into the mountain. Similarly, the vintage tourist cabins built in Yellowstone National Park in the United States have also drawn public dissatisfaction for causing landscape fragmentation^4^.

In natural scenic areas, aesthetic harmony refers to the congruous relationship between human-made elements and natural elements in the landscape^5,6^, pursuing the integration and unity of artificial structures and the natural environment^7^. Aesthetic harmony is regarded as the core of high-quality tourism experience and is also recognized as an important situational factor that fosters tourists’ environmental concern and responsible behaviors^5,6^. As the perception core and experience subject of landscape, tourists’ psychological cognition and behavioral decisions are deeply interactively associated with the physical environment of the destination^8^. Therefore, artificial facility intrusion not only directly undermines the aesthetic value of the destination but may also serve as a persistent implicit environmental cue, exerting a profound impact on tourists’ psychology and behavior.

According to the Broken Window Theory, explicit disorder or destruction in the environment, such as litter and graffiti, can signal the loosening of norms and induce more subsequent illegal behaviors^9^. By inference, highly intrusive artificial facilities that are incompatible with the natural environment may also constitute a static and systematic “visual broken window”, conveying similar negative environmental cues to tourists. Against this background, does such a “visual broken window” similarly trigger the broken window effect at the psychological and behavioral levels, thereby inhibiting tourists’ pro-environmental behaviors? Furthermore, the sound operation and sustainable development of scenic areas and other tourism destinations rely on tourists’ voluntary cooperation and behavioral compliance^10^. Could the intrusion of artificial facilities thus erode the psychological foundation of tourists’ participation in environmental protection, and consequently pose a potential threat to the long-term conservation and sustainable development of scenic areas?

Accordingly, this study focuses on the practical phenomenon of artificial facility intrusion, aiming to explore its effects on tourists’ pro-environmental behaviors and the underlying mechanism. Using three sequential scenario experiments, this study verifies the negative impact of artificial facility intrusion on tourists’ pro-environmental behaviors and the chain mediation via the aesthetic experience–place attachment chain. Meanwhile, it reveals the moderating role of ecological environment value in the transformation from aesthetic experience to place attachment. This study intends to provide theoretical support and practical implications for the planning and management of natural scenic areas, the improvement of tourist experience, and the achievement of sustainable development.

## Literature review

### Artificial facility intrusion

The concept of intrusion has been applied across diverse disciplines, including sociology^11^, psychology^12^, organizational behavior^13^, cybersecurity^14^, and ecology^15^. Although its specific connotations vary across disciplinary perspectives, the core meaning centers on crossing a certain boundary or limit to enter a space, system, or state that does not originally belong to oneself. Intrusion is not merely a physical event but also a psychological and social cognitive process. It triggers individuals’ perception of “order”^11^, may generate resource consumption and stress^13^, and can also elicit or inhibit specific adaptive or maladaptive behaviors^13^. Derived from this core connotation and extended to the context of natural environments in tourism, artificial facility intrusion refers to the “boundary-crossing” and “disruption” imposed on tourists’ expected environmental order, aesthetic experience, and sense of place by excessive, inappropriate, or poorly maintained artificial structures and their accompanying visual, ecological, or spatial disturbances in natural tourism settings.

To meet the demands of tourism development, various artificial facilities are inevitably introduced or constructed within natural landscapes, and such construction constitutes an intervention in and transformation of the original natural state of the landscape^2^. Although existing studies have examined how human-made facilities—including roads^16^, buildings, renewable energy facilities^17^, and wind energy facilities^18^—affect the aesthetic value of regional environments and cause visual pollution, prior research has mostly focused on the visual disturbance and aesthetic damage caused by large‑scale, engineered artificial facilities in macro natural spaces such as wilderness areas and ecological protection zones. Moreover, investigations into the impacts of artificial facilities have largely centered on macroscopic visual conflicts and ecological destruction. However, few studies have focused specifically on natural scenic areas as a distinctive tourism spatial context, nor have they systematically explored the impacts of small‑scale, routinely supporting artificial facilities on the natural environment within such destinations. Although artificial facilities built in natural scenic areas for tourism development are relatively small and more scattered in layout, they represent environmental elements frequently encountered by tourists during their travel experiences. In tourism practice, these facilities often result in subtle intrusion into natural scenic spaces due to inadequate planning, random placement, and design disconnection from the natural landscape. Such intrusion does not appear as explicit large‑scale ecological destruction, yet it imperceptibly disrupts the continuity of the natural landscape and diminishes the natural aesthetic characteristics of scenic areas, thus becoming an important subtle factor constraining the sustainable development of scenic areas. Therefore, it is necessary to examine the impact mechanism of artificial facility intrusion in natural scenic areas.

### Aesthetic experience and aesthetic harmony

Aesthetic experience refers to the unique perceptual experience generated by the aesthetic subject when observing and perceiving the aesthetic object under the synergistic effect of physiological perception and psychological cognitive mechanisms. Experience is a joint product of the interaction between the aesthetic subject and the object in psychological activities. In short, aesthetic experience is the result of the combined effect of physiological and psychological mechanisms, which reflects the complex and subtle interaction between the subject and the object^19^ Experience is the core of tourism activities, and as a special aesthetic process^20^, tourism experience is closely linked to landscape aesthetics. Existing studies have identified five key aesthetic dimensions of natural scenic areas, including harmony, change/contrast, scenery/viewing, authenticity, and art/architecture, which collectively construct tourists’ aesthetic perception framework of natural landscapes^7^. Among these dimensions, aesthetic harmony mainly reflects the harmonious relationship between human-made and natural elements in the landscape^5,6^. It emphasizes the harmonious unity of artificial structures and natural landscapes, enabling tourists to get close to nature in depth and have a sense of integration with nature^7^. Aesthetic harmony requires the landscape to possess coherence^21^, unity^22^, and coordination. And as the core of aesthetic dimensions, it is conducive to the formation of high-quality aesthetic experience. Tourism destinations with good aesthetic quality can bring pleasant aesthetic emotions to tourists, thereby enhancing their satisfaction, loyalty, and revisit intention^23^. In addition, a good aesthetic experience can trigger tourists’ emotional resonance (Breiby and Slåtten^76^), which in turn stimulates their environmental protection awareness and behaviors, in which the place attachment plays an important role. High-quality aesthetic experience helps form the place attachment—a strong interaction between humans and the environment that contributes to enhancing people’s care and support for the environment^24^. Strong place attachment makes people less willing to tolerate environmental damage caused by tourism and promotes them to participate more actively in environmental protection^25^. Therefore, destinations that provide high-quality aesthetic experience can promote tourists’ pro-environmental behaviors.

Based on the above reviews, existing studies have mostly focused on aesthetic harmony and its positive outcomes, with limited attention paid to the occurrence and potential impacts of aesthetic disharmony. In tourism practice, however, aesthetic disharmony is prevalent, among which artificial facility intrusion serves as a typical manifestation. It undermines the visual integrity and aesthetic harmony of destinations, posing threats to tourists’ aesthetic experience, destination image, and sustainable development. Such problems triggered by aesthetic disharmony are particularly acute and complex in natural scenic areas. For natural landscapes, preserving the authenticity of nature and providing tourists with high-quality aesthetic experience are especially crucial. From an aesthetic perspective, a landscape is a spatial combination of elements that delivers subjective aesthetic pleasure and satisfaction to individuals, representing the integration of natural and cultural phenomena under human visual perception^26^. When the form and features of a natural landscape are altered by the intrusion of artificial facilities, visual pollution occurs, causing emotional and psychological discomfort among tourists, and even evoking inner confusion and dissatisfaction, thereby inducing a negative chain reaction like the broken window effect^2^. Thus, artificial facility intrusion not only directly damages the aesthetic harmony and experience of natural landscapes but also conveys negative signals through visual and other sensory channels, exerting adverse impacts on tourists’ psychology and behaviors. Furthermore, previous studies have mainly examined the relationships of aesthetic experience, aesthetic harmony and satisfaction, revisit intention, while insufficient attention has been paid to altruistic behaviors such as pro-environmental behaviors. Therefore, it is necessary to further investigate how artificial facility intrusion influences tourists’ pro-environmental behaviors through aesthetic disharmony and to reveal the underlying psychological mechanisms. This study aims to fill the research gap and provide theoretical implications for the planning and management of natural scenic areas.

### Pro-environmental behavior

Pro-environmental behavior refers to individuals’ conscious management of their own behaviors, which aims to reduce adverse impacts on the environment or promote positive environmental improvement^27^. Scholars have conducted extensive research on the factors influencing tourists’ pro-environmental behaviors. Overall, these influencing factors mainly include external contextual factors and individual psychological factors. External contextual factors consist of social factors, such as religious identity, urban–rural differences, social class, and social norms^28^, as well as external intervention measures, such as convenience^29^, activity participation^30^, and tourism interpretation systems^31^. However, current research on external contextual influencing factors is still in the preliminary exploration stage, and a systematic theoretical explanatory framework has not yet been formed. At the individual psychological level, the intertwined effects of cognitive and affective factors are prominent. From the cognitive perspective, it is emphasized that pro-environmental behavior stems from individuals’ rational thinking and judgment^32^. The Theory of Reasoned Action (TRA) and the Theory of Planned Behavior (TPB) suggest that attitude, subjective norm, and perceived behavioral control jointly shape behavioral intention, which in turn determines actual behavior. Individuals with positive environmental attitudes are more likely to engage in pro-environmental behaviors^33^. From the affective perspective, it is highlighted that, within the context of tourism immersive experiences, pro-environmental behavior relies primarily on affective pathways rather than rational principles^34–36^. In addition, affective factors such as emotion^37^, satisfaction, place attachment^38–40^, ecologically responsible attitudes^41^, and subjective well-being^42^ have been recognized as critical drivers of pro-environmental behavior. Furthermore, existing studies of psychology on tourists’ pro-environmental behaviors have mostly focused on core internal psychological variables such as cognitive evaluation and emotional bonding and emphasized their direct or indirect impacts of tourists’ subjective psychology on their pro-environmental behaviors. However, insufficient attention has been paid to the underlying psychological mechanisms through which artificial facility intrusion—an objective environmental factor in natural tourism settings—triggers the broken window effect and subsequently influences tourists’ pro-environmental behaviors. Accordingly, against the current context of promoting ecological civilization and sustainable tourism, it is particularly necessary to systematically examine the influencing mechanism of artificial facility intrusion on tourists’ pro-environmental behavior. This will not only broaden the understanding of external situational factors and provide an integrated dynamic framework for understanding tourists’ pro-environmental behavior that incorporates environment, psychology, and behavior, but also offer critical practical insights for destination planning and management.

By exploring how artificial facility intrusion triggers the “broken window” behavior among tourists, managers can conduct facility planning and maintenance more effectively, block the transmission path from environmental disorder to behavioral deviance, and thus more scientifically stimulate and safeguard tourists’ pro-environmental behavior.

## Research hypotheses

### The impact of artificial facility invasion on tourists’ environmentally friendly behaviors

Pro-environmental behavior refers to actions taken by individuals or groups that can minimize harm to the natural environment and contribute to environmental protection^43^. For a long time, exploring the influencing factors of pro-environmental behavior has been an important research topic in the field of natural tourism. Affective factors are important drivers of pro-environmental behavior and have attracted increasing attention from researchers. According to the Stimulus-Organism-Response (SOR) model, external environmental factors affect human behavior by acting on their psychological perceptions. In the tourism context, the aesthetic characteristics of destinations can trigger emotional responses^44^, enhance personal preferences^45^, and promote positive consumer behaviors^46^. Thus, the visual harmony of landscapes can bring high-quality aesthetic experiences to tourists, enhance their pleasure, strengthen their place attachment, reduce their tolerance for environmental damage^34^, and motivate their positive pro-environmental behaviors. Conversely, when negative environmental stimuli such as cluttered signs, excessive advertisements, or incompatible artificial facilities appear in natural scenic areas^47^, they will cause tourists to experience physiological stress and discomfort, making them more likely to engage in illegal behaviors such as environmental damage ^47^. Like the “visual broken window”, the visual disharmony of landscapes caused by artificial facility intrusion can also trigger negative behavioral responses among tourists, such as inducing more environmental damage behaviors ^48^. Therefore, we propose H1.

#### H1

The artificial facility intrusion has a significant negative impact on pro-environmental behavior. Compared with low-degree artificial facility intrusion, high-degree artificial facility intrusion will more strongly inhibit tourists’ pro-environmental behaviors.

### The impact of artificial facility intrusion on aesthetic experience

Artificial facility intrusion refers to compulsory, heterogeneous intervention, disturbance and even destruction imposed by human-made structures (i.e., artificial facilities) constructed to meet specific human functional needs on the integrity, harmony, and aesthetic experience of a native or existing natural and cultural landscape system. Existing research shows that landscapes without artificial elements are perceived as aesthetically harmonious^49^. Natural landscapes containing artificial elements tend to attract greater visual attention. The higher the visual sensitivity of a landscape, the more visual pollution is imposed on tourists, and the higher the degree of landscape disharmony^50–53^.

Natural landscapes possess high aesthetic value due to their ecological characteristics^54^. However, artificial facilities in natural landscapes undermine the visual integrity and aesthetic harmony of the landscape, resulting in intense visual conflict and aesthetic pollution^47^. Because visual pollution impairs the visual quality and aesthetic harmony of the landscape and violates tourists’ aesthetic expectations formed through environmental aesthetics, it may trigger aesthetic dissonance^55^. Therefore, we propose H2.

#### H2

The degree of artificial facility intrusion has a significant negative effect on aesthetic experience.

### The effect of aesthetic experience on place attachment

When visiting landscapes, tourists’ aesthetic responses are often regarded as aesthetic experiences that underpin aesthetic value^56^, which are described as aesthetic affections in response to sensory aesthetic qualities^57^. Aesthetic experience can optimize tourists’ overall experience and influence their pleasant experiences and positive emotions^23,46^. Therefore, aesthetic experience can not only optimize the overall perceived quality of the visit but also directly generate positive emotions such as pleasure and immersion among tourists.

Place attachment refers to a deeper bond formed in the interaction between individuals and place environment. Individuals’ perception of the environment is a critical prerequisite for the formation of place attachment; through perception, people establish a close connection with the environment, thereby developing affective, cognitive, and behavioral dependence on and identification with a place. The quality of environmental conditions significantly influences individuals’ psychological states and behavioral responses^58^. A visually appealing landscape can evoke and strengthen tourists’ emotional attachment to a place^59^, which in turn fosters place attachment. At its core, the formation of place attachment relies on the establishment of a stable and profound emotional bond between individuals and the place. This bond largely stems from the positive affective experiences gained within a specific setting. When tourists perceive a harmonious natural landscape during their visit, develop a deep aesthetic identification and emotional resonance, and consequently attain a pleasurable and immersive aesthetic experience, they experience stronger positive emotions and psychological satisfaction. Such internal positive affective experiences further reinforce tourists’ favorable cognitive evaluations and emotional preferences toward the destination, driving the continuous deepening of the emotional connection between tourists and the place, and ultimately facilitating the gradual formation and sustained enhancement of place attachment^60^. Therefore, we propose H3.

#### H3

Aesthetic experience has a significant positive effect on place attachment.

### The effect of place attachment on pro-environmental behavior

The emotional bond between individuals and destinations is rooted in place attachment theory. Place attachment refers to an emotional connection that individuals are likely to form with a destination when they acquire positive experience in the tourism context^27,61^. Therefore, place attachment helps explain tourists’ travel intention, satisfaction, loyalty, and pro-environmental behavior toward the destination^62–65^. For example, place attachment can positively influence pro-environmental behavior^66^. The higher the level of place identity, the stronger an individual’s tendency to protect the local environment, such as picking up litter or participating in related environmental activities. Place attachment serves as an important antecedent variable of pro-environmental behavior (Anton and Lawrence^79^; Scannell and Gifford^78^). It enables individuals to endow a place with positive feelings, meaning, and value, and strengthens tourists’ emotional bond with the place. Such an emotional bond stimulates tourists’ sense of place responsibility and belonging and prompts them to shift from a “bystander” perspective to a “stakeholder” perspective. They will actively care about the ecological sustainability of the scenic area, weaken self-serving tendencies, and strengthen the awareness of the environment. Thus, when people are in the natural environment, place attachment helps enhance their care and support for the environment. Strong place attachment makes people less tolerant of environmental damage caused by tourism and strengthens their willingness to safeguard the interests of the natural environment^25^. Therefore, we propose H4.

#### H4

Place attachment has a significant positive effect on pro-environmental behavior.

### The moderating role of ecological environment value

**Fig. 1:**
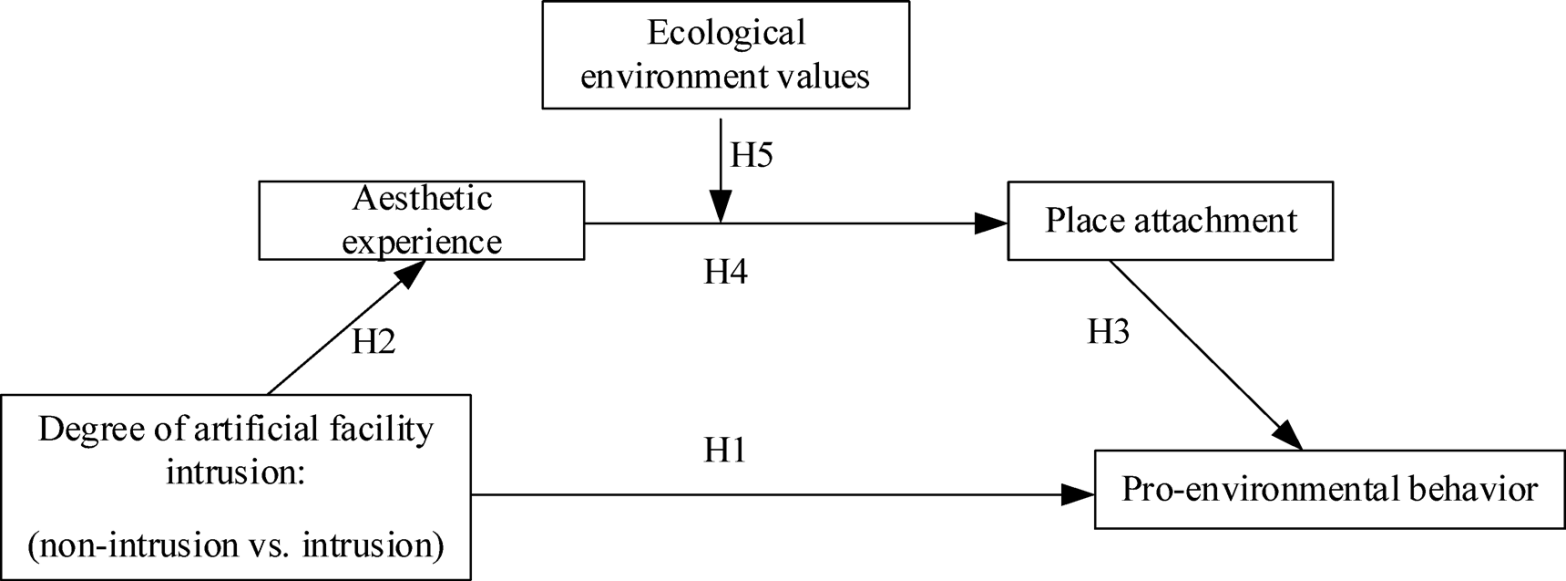
Diagram of the theoretical model.

The Value-Belief-Norm (VBN) theory posits that values, attitudes, and beliefs play a leading role in social norms and responsibilities, which in turn influence participation of specific behaviors, such as pro-environmental behavior and environmentally responsible behavior^67^. As the starting point of the VBN theory model, ecological environment value refers to the values individuals perceive regarding the environment and related issues^68^. They reflect individuals’ core attitudes and views on the natural environment and related issues based on their own philosophy of life, embodying individuals’ in-depth cognitive and emotional responses to environmental problems^69^. Ecological environment value includes individuals’ understanding of the environment and evaluation of environment values, such as the importance of the environment to ecosystem balance, the assessment of the impact of human behaviors on the environment, and whether certain behaviors promote the harmonious coexistence between humans and nature. The aesthetic experience generated by individuals in their interaction with a specific regional environment is essentially a subjective psychological experience formed by the interaction between elements such as the environment’s natural landscape, ecological characteristics, and cultural connotations and individuals’ perceptual systems. It can bring sensory and physical pleasure to tourists and trigger positive emotional responses^70^. Ecological environment value, however, endows this perceptual experience with richer ecological value connotations, enabling individuals to go beyond the perception of the superficial visual beauty of the environment, deeply understand the ecological functions, system integrity, and survival significance of the environment, and elevate aesthetic experience from pure sensory pleasure to an in-depth experience that combines ecological cognition and emotional identification. This aesthetic experience endowed with ecological value cognition will further strengthen individuals’ affective investment and psychological identification with the local environment^71^. At the same time, ecological environment value shapes individuals’ value orientation and emotional connection mode with the local environment. Individuals with ecological environment value will link their aesthetic experience with their sense of responsibility for protecting the local environment and emotional belonging, enabling them to easily generate a sense of affection, belonging, and identification with the local environment while gaining aesthetic pleasure^72^. Therefore, we propose H5 (see Fig. 1).

#### H5

Ecological environment value plays a positive moderating role in the relationship between aesthetic experience and place attachment. Specifically, the higher an individual’s level of ecological environment value, the stronger the positive effect of aesthetic experience on place attachment; conversely, this positive effect will be weakened.

## Methodology

This study sequentially examines the main effects, mediating effects, and moderating effects through three studies to explore the mechanism and boundary conditions of the impact of artificial facility intrusion on individuals’ pro-environmental behaviors in natural tourism destinations (Table 1). Specifically, Study 1 employed an experimental situational method to preliminarily verify the impact of artificial facility intrusion on pro-environmental behaviors. Study 2, based on Study 1, further verified the mediating effects of aesthetic experience and place attachment by changing the stimulus materials and experimental scenarios. Finally, Study 3 replaced the stimulus materials and experimental scenarios again and examined the moderating effect of ecological environment value.

**Table 1:** Study design.

Examine	Hypotheses	Experimental design	Methodologies
Study 1	Main effect: H1	Degree of artificial facility intrusion: non‑intrusion vs. intrusion	Situational experiment
Study 2	Mediating effects: H2, H3, H4	Degree of artificial facility intrusion: non‑intrusion vs. intrusion	Situational experiment
Study 3	Moderating effect: H5	2 (Degree of artificial facility intrusion: (non‑intrusion vs. intrusion) × 2 (Ecological environment value: low vs. high)	Situational experiment

### Study 1

#### Research design

Study 1 used a single-factor (degree of artificial facility intrusion: non‑intrusion vs. intrusion) between-subject design to initially verify the effect of the degree of artificial facility intrusion on tourists’ willingness to engage in pro-environmental behaviors. Study 1 selected the glass viewing platform in the Shilin Gorge Scenic Area in Beijing, China as the experimental site. This viewing platform is supported by a huge titanium alloy arch structure, forming a circular transparent glass platform suspended above the peak of the canyon. Its visually striking artificial architectural form creates a strong aesthetic contrast with the surrounding natural canyon, reservoir, and forest landscapes. In the experimental design, the DBAOI software was used to standardize the original images, removing the artificial facility of the flying saucer-shaped glass viewing platform from the picture. At the same time, the original landscape elements such as the shooting angle, lighting parameters, color, and terrain vegetation were strictly maintained unchanged, to accurately separate and observe the impact of the target artificial facility and minimize the interference of irrelevant variables to ensure the rigor of the experimental design.

**Fig. 2:**
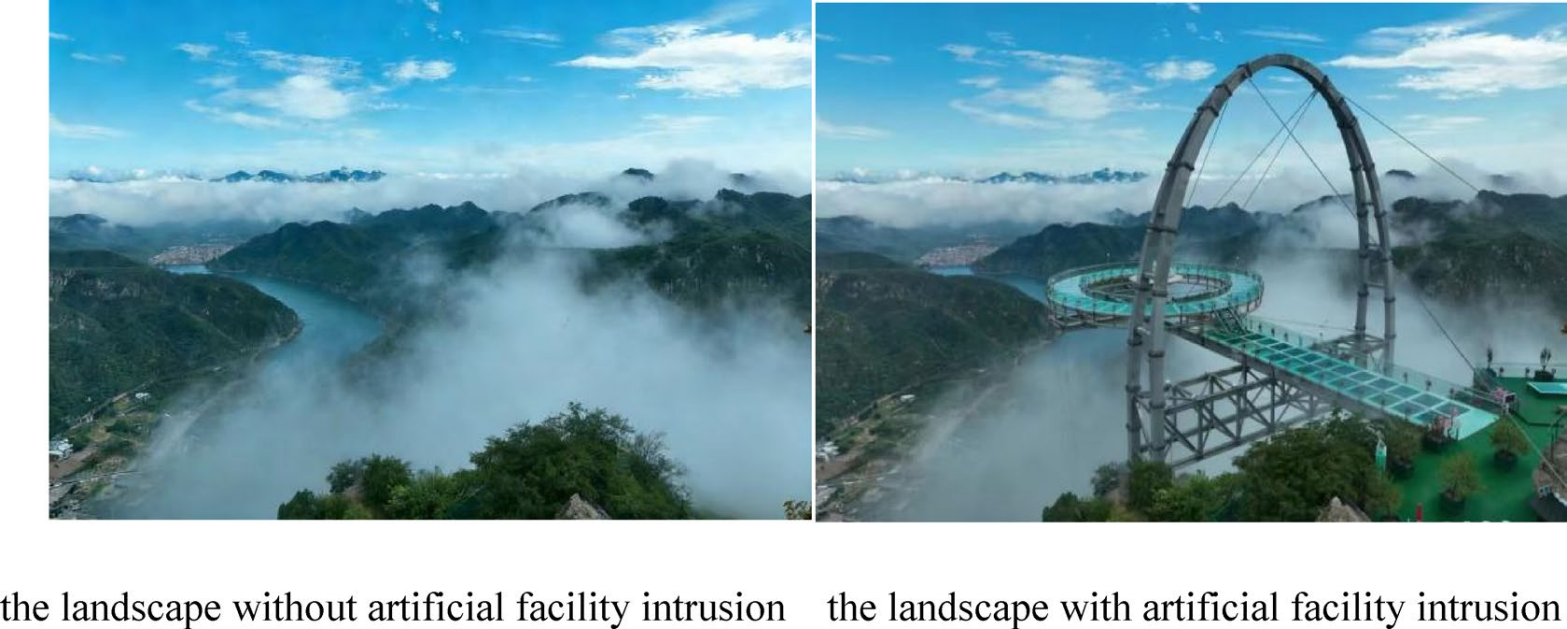
Experimental materials of Study 1.

To ensure the validity of the experimental manipulation, this study first set up two natural landscape experimental scenarios: “artificial facility intrusion” and “no artificial facility intrusion” and conducted a pre-test. The core purpose was to verify that there was a significant difference in the perceived degree of artificial facility intrusion between the two scenario groups. Among them, the artificial facility intrusion group used the original landscape pictures of the Beijing Stone Forest Gorge scenic area with the flying saucer glass viewing platform; the group without artificial facility intrusion removed the viewing platform through standardized digital editing technology, while preserving all other natural elements (see Fig. 2). The artificial facility intrusion was measured using the scale designed by Kotabe et al.^48^, which measured through a total of 4 items (α = 0.808), such as “This landscape is highly coordinated with the surrounding environment, and the overall aesthetic is naturally unified” and “The landscape harmoniously integrates with the surrounding natural scenery, without any incongruity”, using a 7-point rating (1 = strongly disagree, 7 = strongly agree).

The survey questionnaire was released through the Credamo platform. The research purpose, participation duration and informed consent matters will be clearly stated in the questionnaire. Participants who completed the filling would be rewarded with 1 yuan. A total of 60 participants were recruited for the pre-test experiment (56.70% female, M_age_ = 31.27, SD = 5.801). The results showed that for the case of artificial facility intrusion, the performance of the group with artificial facility intrusion was significantly higher than that of the group without artificial facility intrusion. (M_non‑intrusion_ = 3.67, SD = 0.877; M_intrusion_ = 5.22, SD = 0.659; F (1, 58) = 59.919, *p* < 0.001, η^2^ = 0.508). Therefore, the experimental material met the need for experimental manipulation.

142 participants (50.0% female, M_age_ = 31.96, SD = 7.255) recruited for the formal experiment were randomly assigned to one of two experimental groups. First, a unified scene introduction text was provided to participants in both groups, describing the Shilin Xia Flying Saucer Glass Observation Platform and the surrounding canyon-reservoir landscape. This scenic area is a multi-functional mountainous scenic area located in Huangsongyu Township, Pinggu District, Beijing, based on the granite canyon of Shilin Xia and the Huangsongyu Reservoir. The surrounding natural setting is characterized by canyon peaks and temperate deciduous broad-leaved forests, with frequent cloud formations in summer. Participants were instructed to imagine themselves being on a trip to this destination and to complete a series of questions after “finishing” the trip. At the end of the text presentation, photographic materials (see Fig. 2) were displayed to participants in both groups.

Participants were then asked to complete a questionnaire. The measurement items of artificial facility intrusion were consistent with those used in the pre-test (α = 0.889). The indicators for measuring pro-environmental behavior were adapted from the scale developed by Smith-Sebasto et al.^73^, with appropriate adjustments and optimizations made to align with the context of this study. Example items included “If there is no trash can, I will carry my own trash with me” and “I will actively dissuade or stop other tourists from damaging the environment,” totaling 4 items (α = 0.856). All scales adopted a 7-point Likert scale (1 = strongly disagree, 7 = strongly agree). Finally, demographic information about participants (e.g., gender, age) was collected.

#### Findings

***Manipulation checks***. A one-way analysis of variance (ANOVA) was conducted to verify the effectiveness of the manipulation. The results indicated that regarding the degree of artificial facility intrusion, the non-intrusion group scored significantly lower than the intrusion group (M_non-intrusion_ = 3.48, SD = 0.548; M_intrusion_ = 5.27, SD = 0.572; F(1, 140) = 363.367, *p* < 0.001, η^2^ = 0.722). These results confirmed that the manipulation of artificial facility intrusion was successful.

**Fig. 3:**
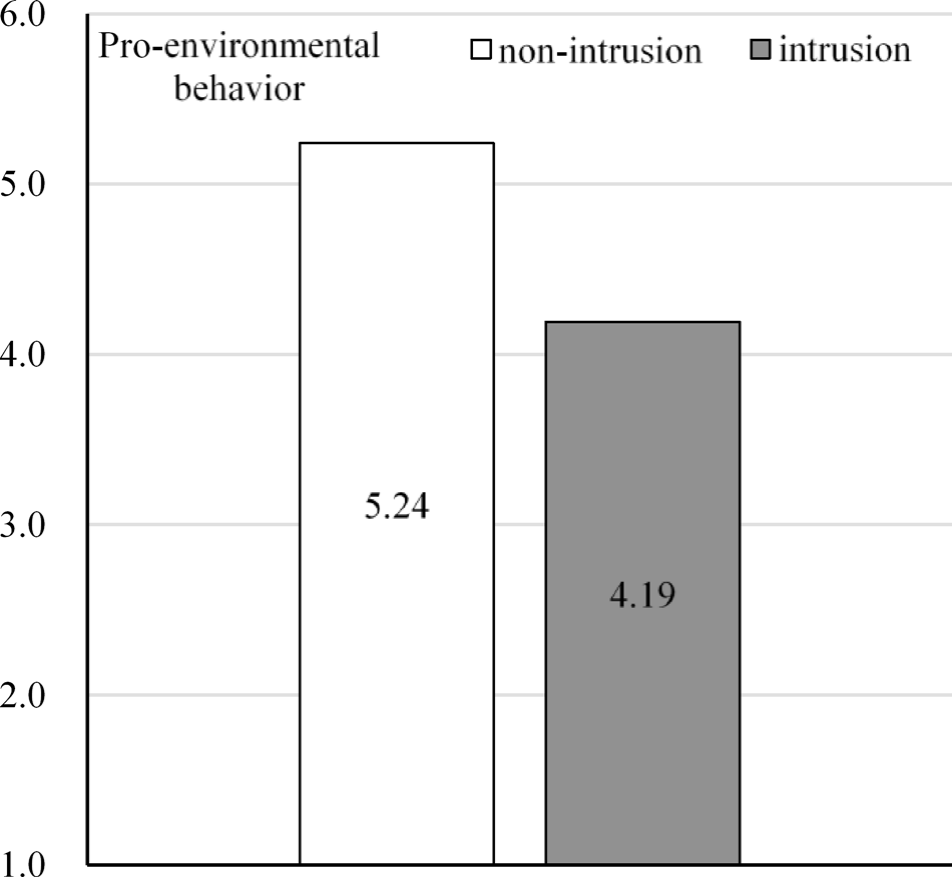
Results of study 1.

***Main effects***. A one-way analysis of variance (ANOVA) was performed to examine the main effect of artificial facility intrusion on tourists’ pro-environmental behaviors. The results indicated that tourists’ pro-environmental behavior scores in the no-intrusion group were significantly higher than those in the intrusion group (M_non-intrusion_ = 5.24, SD = 0.940; M_intrusion_= 4.19, SD = 0.60; F(1, 140) = 62.843, *p* < 0.001, η^2^ = 0.310) (see Fig. 3). Specifically, compared with landscapes with artificial facility intrusion, tourists exposed to landscapes without such intrusion were more willing to engage in environment-friendly behaviors. These results provided support for Hypothesis 1 (H1).

#### Discussion

Study 1 initially conducted as situational experiment to test the hypothesis that artificial facility intrusion in natural tourist destinations would inhibit tourists’ pro-environmental behaviors. Although this study preliminarily revealed the relationship between artificial facility intrusion and pro-environmental behaviors, the underlying psychological mechanisms driving the effect remain unclear. Therefore, further experiments are required to deeply explore how artificial facility intrusion elicits tourists’ psychological cognitive and emotional responses and subsequently inhibits their pro-environmental behaviors.

### Study 2

#### Research design

Study 2 also adopted a single-factor (degree of artificial facility intrusion: non-intrusion vs. intrusion) between-subject design. Based on retesting the findings of Study 1, this study modified the experimental scenario to examine the mediating mechanism underlying the main effect. The destination selected for Study 2 was the Dagu Glacier Scenic Area in Sichuan Province, China, which boasts a unique and representative snow-capped mountain natural landscape. Meanwhile, its relatively few traces of tourism development provided a relatively pure experimental context for the study, helping to minimize the impact of external interfering factors on the experimental results.

**Fig. 4:**
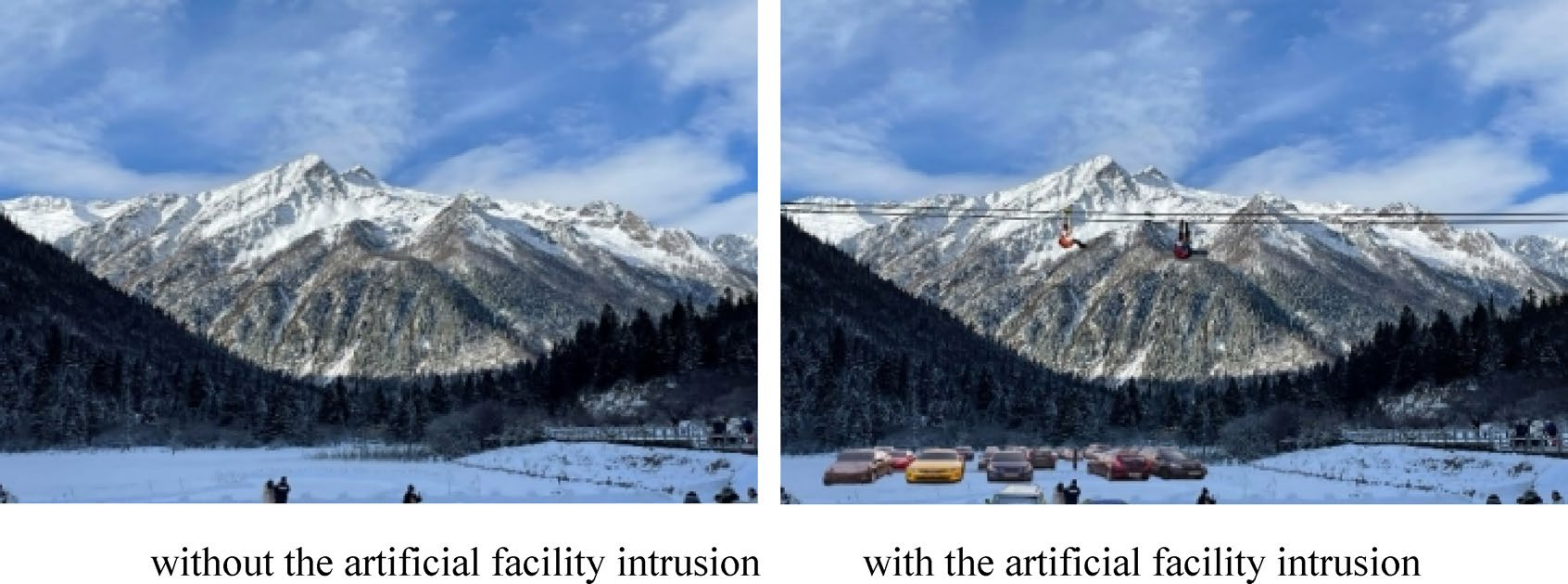
Experimental materials of Study 2.

Similarly, a pretest of artificial facility intrusion was conducted using natural landscape stimuli with or without artificial facility intrusion. The recruitment procedure was identical to that in Study 1. Photographs in the no‑intrusion group depicted a remote ice peak in Dagu Glacier Park, whereas those in the intrusion group added artificial facilities such as zip lines and parking lots to the same scene, with all other elements remaining unchanged (see Fig. 4). Artificial facility intrusion was measured using the same procedure and scale adopted in Study 1^48^, α = 0.888). The pretest recruited 60 participants (59.0% female, M_age_ = 31.08, SD = 6.639). The results revealed that perceived artificial facility intrusion was significantly higher in the intrusion condition than in the no‑intrusion condition (M_non-intrusion_ = 4.21, SD = 0.546; M_intrusion_ = 5.34, SD = 0.466; F(1, 58) = 74.768, *p* < 0.001, η^2^ = 0.563). Thus, the experimental materials were deemed suitable for experimental manipulation.

A total of 120 participants (43.7% female, M_age_ = 30.62, SD = 5.754) were recruited for the formal experiment and randomly assigned to one of two conditions. The participant recruitment procedure was identical to that in Study 1. First, a uniform scenario introduction was administered to participants in both conditions. Specifically, Dagu Glacier is in Aba Prefecture, Sichuan Province, and is a natural ecological tourist destination characterized by glaciers, snow-capped mountains, and forests. Participants were instructed to imagine traveling to the glacier according to the information in the subsequent pictures and to complete relevant questions after the virtual tour. Following the text introduction, the picture stimuli (see Fig. 4) were presented to both groups in the same manner. The two pictures were identical in terms of landscape composition, perspective, and brightness, with the only difference being the presence of artificial facilities.

Participants were then asked to complete a questionnaire. The measurement items for artificial facility intrusion and pro-environmental behavior were identical to those used in Study 1. Aesthetic experience was measured using a scale adapted from Chen et al.^74^ and Leder et al.^75^, consisting of six items. Example items included “This landscape brings me visual pleasure,” “When I saw this scene, I felt a sense of relaxation and calmness inside,” “When I saw this scene, I felt extremely happy,” and “When I saw this scene, I felt both physically and mentally content.” All items were rated on a 7-point Likert scale (1 = strongly disagree, 7 = strongly agree). Place attachment was measured using a well-established scale developed by Halpenny^61^, with six items. Example items included “I have a strong sense of belonging here” and “This landscape holds great significance for me.” These items were also rated on a 7-point scale (1 = strongly disagree, 7 = strongly agree). Finally, participants’ demographic information (e.g., gender, age) was collected.

#### Findings

***Manipulation checks.*** A one-way ANOVA was conducted to test the effectiveness of the experimental manipulation. The results indicated that the degree of artificial facility intrusion in the no‑intrusion group was significantly lower than that in the intrusion group (M_non-intrusion_ = 2.65, SD = 1.045; M_intrusion_ = 4.82, SD = 0.785; F(1, 118) = 165.316, *p* < 0.001, η^2^ = 0.584). These results confirmed that manipulation was successful.

**Fig. 5:**
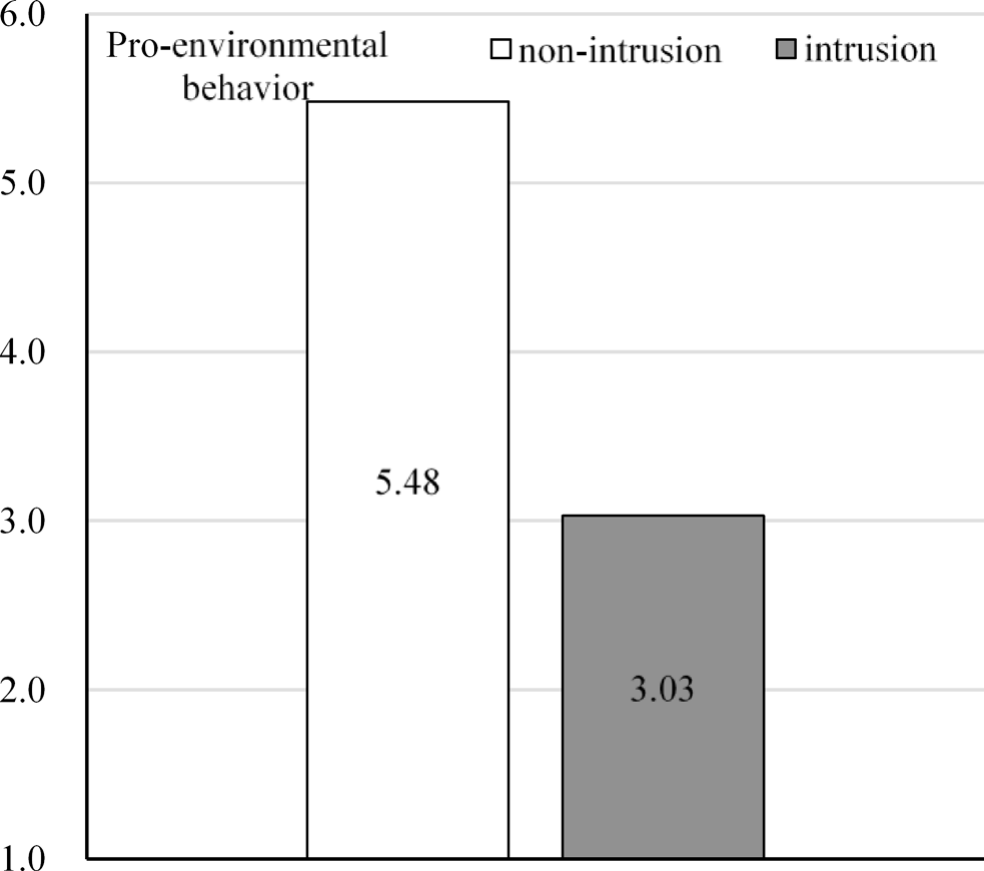
Main effects intergroup results of Study 2.

***Main effects.*** A one-way ANOVA was conducted to examine the main effect of artificial facility intrusion on tourists’ pro-environmental behavior. The results indicated that pro-environmental behavior was significantly higher in the no-intrusion group than in the intrusion group (M_non-intrusion_ = 5.48, SD = 0.90; M_intrusion_ = 3.03, SD = 0.727; F(1, 118) = 268.161, *p* < 0.001, η^2^ = 0.694) (see Fig. 5). Specifically, compared with landscapes affected by artificial facility intrusion, tourists exposed to unspoiled landscapes were more willing to engage in pro-environmental behaviors. These results provided full support for Hypothesis 1 (H1).

***Mediating effects.*** A one-way ANOVA was conducted to examine the effect of artificial facility intrusion on aesthetic experience. The results indicated that there was a significant difference in aesthetic experience between the two groups (non-intrusion vs. intrusion) (M_non-intrusion_ = 5.22, SD = 0.862; M_intrusion_ = 2.91, SD = 0.652; F(1, 118) = 273.872, *p* < 0.001, η^2^ = 0.699). Specifically, aesthetic experience scores in the no-intrusion group were significantly higher than those in the intrusion group.

**Fig. 6:**
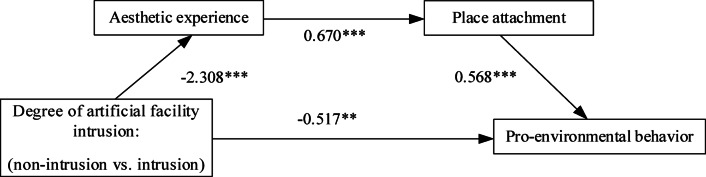
Results of Study 2.

The chain mediating effect of aesthetic experience and place attachment was tested using the Bootstrap method (PROCESS, Model 6, n = 5000). The results revealed that when pro-environmental behavior was the dependent variable, the chain mediating effect of aesthetic experience and place attachment was significant (β =  − 0.881, LLCI =  − 1.297, ULCI =  − 0.488, 95% CI does not include zero). Results were presented in Table 2. After controlling for the mediating variables, the effect of artificial facility intrusion (non‑intrusion vs. intrusion) on pro-environmental behavior remained significant (β = − 0.517, LLCI = − 0.880, ULCI = − 0.154, 95% CI does not include zero), indicating that aesthetic experience and place attachment played a partially mediating role. The results of Experiment 2 were displayed in Fig. 6. In addition, aesthetic experience exerted a significant positive effect on place attachment (β = 0.670, LLCI = 0.500, ULCI = 0.840, 95% CI does not include zero), and place attachment positively predicted pro-environmental behavior (β = 0.568, LLCI = 0.431, ULCI = 0.705, 95% CI does not include zero). Therefore, Hypotheses H2, H3, and H4 were supported.

**Table 2:** Mediating effects of aesthetic experience and place attachment orientation.

Effect type	Effect	SE	t	Sig	95% CI	
					LLCI	ULCI
Direct effect	− 0.517	0.183	− 2.822	0.006	− 0.880	− 0.154
Indirect effect						
IV → AE → PB	− 0.619	0.252			− 1.097	− 0.122
IV → PA → PB	− 0.427	0.133			− 0.717	− 0.195
IV → AE → PA → PB	− 0.881	0.207			− 1.297	− 0.488

IV is the independent variable of degree of artificial facility intrusion (non‑intrusion vs. intrusion), AE is aesthetic experience, PA is place attachment, PB is pro-environmental behavior.

#### Discussion

Study 2 reveals the mediating mechanism through which aesthetic experience and place attachment operate. This study demonstrates that natural landscapes without artificial facility intrusion can enhance aesthetic experience, thereby stimulate tourists’ positive emotions and high satisfaction, and strengthen their emotional attachment to the destination. Such intensified emotional attachment prompts tourists to be more willing to engage in environmentally friendly actions from the perspective of the destination, pay close attention to environmental issues, and recognize their moral responsibility toward the environment.

Although this mediating mechanism has been clearly identified, its applicable conditions and boundary factors still require further investigation. Therefore, Study 3 focuses on examining the moderating role of ecological environment value, aiming to further clarify the complex relationship between artificial facility intrusion and pro-environmental behavior.

### Study 3

#### Research design

Study 3 adopted a 2 (artificial facility intrusion: non‑intrusion vs. intrusion) × 2 (ecological environment value: low vs. high) between‑groups experimental design. The experimental scenario was revised in Study 3, and Qiaxi Forest Park in Xinjiang, China, was selected as the research site. Qiaxi Forest Park is in the core area of the Tianshan Mountains and constitutes an important part of the Western Tianshan Natural Heritage Site. It is famous for its well‑preserved primeval spruce forests and magnificent mountain‑forest‑steppe vertical band spectrum landscape. As a key ecotourism destination in the Ili River Valley, it attracts numerous tourists for ecological sightseeing and forest hiking. However, as a typical ecologically fragile region, this area is facing environmental pressure caused by the expansion of tourism activities and potential climate change. Consequently, the protection of the authenticity and integrity of its natural ecosystem deserves particular attention.

**Fig. 7:**
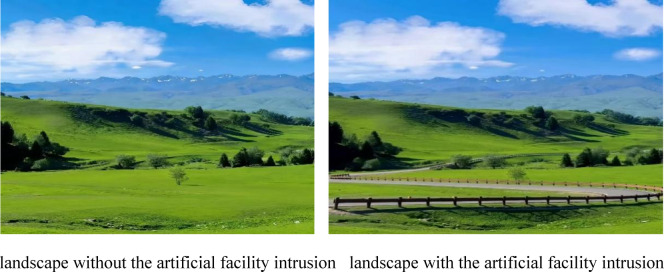
Experimental materials of Study 3.

First, a pretest of artificial facility intrusion was conducted using landscape stimuli depicting the non‑intrusion and intrusion conditions. Photographs for the intrusion group showed landscapes with visible artificial roads and fences within Qiaxi Forest Park in Xinjiang. For the non‑intrusion group, roads and fences were removed from the original scene, with all other elements remained unchanged (see Fig. 7). Artificial facility intrusion was measured using the same procedure and scale as in Study 1^48^, α = 0.863). The recruitment procedure was identical to that in Study 1. A total of 58 participants took part in the pretest (56.90% female, M_age_ = 25.88, SD = 3.298). The results revealed that perceived artificial facility intrusion was significantly higher in the intrusion group than in the non‑intrusion group (M_intrusion_ = 5.79, SD = 0.819; M_non-intrusion_ = 1.59, SD = 0.946; F(1, 56) = 119.264, *p* < 0.001, η^2^ = 0.854). Thus, the experimental materials were deemed appropriate for experimental manipulation.

A pretest was conducted to validate the manipulation of ecological environment value, using two different written materials describing Qiaxi Forest Park in Xinjiang, China.

For the group with high ecological environment value, the material emphasized the intrinsic value of the ecosystem and the moral obligation for protection: *“As an important part of the West Tianshan Heritage Area, Qiaxi Forest Park’s well-preserved primeval spruce forests and complex mountain vertical zonation possess unique aesthetic and scientific value and are crucial for maintaining regional ecological balance and biodiversity. Protecting such primeval ecosystems is a moral obligation to the integrity of natural heritage itself, transcending humans’ practical benefits. As part of the ecosystem, humans are responsible for preserving its natural processes and authenticity through minimally intrusive means, ensuring it can be enjoyed by future generations.”*

For the group with low ecological environment value, the material focused on the utilitarian and functional values of natural resources: *“As an important forest resource and tourist destination, Qiaxi Forest Park’s spruce forests and mountain landscapes play a significant role in water conservation, soil and water conservation, and providing recreational services. The protection of this area primarily aims to sustain its beneficial functions and service capabilities for human society, ensuring the sustainable utilization of resources to support the long-term well-being and development of local communities.”* A total of 60 participants were recruited for this pretest (56.67% female, M_age_ = 24.67, SD = 3.611). The results of a one-way ANOVA indicated that the high ecological environment value group scored significantly higher than the low ecological environment value group (M_low_ = 3.14, SD = 1.026; M_high_ = 5.55, SD = 0.624; F(1, 58) = 122.778, *p* < 0.001, η^2^ = 0.679). These results confirmed that the experimental materials were effective for manipulating ecological environment value.

A total of 373 participants (47.72% female; M_age_ = 26.91, SD = 4.650) were recruited for the formal experiment and randomly assigned to one of four experimental conditions. The participant recruitment procedure was identical to that in Study 1. First, a uniform scenario introduction was administered to all participants: “*Qiaxi Forest Park is in the mountain forest and grassland area of the Ili River Valley in Xinjiang, China. It is a renowned ecotourism destination in the Ili River Valley, attracting numerous visitors each year. Tourists can walk through primeval spruce forests and alpine meadows and observe unique mountain vegetation and wildlife. Please view the following pictures and imagine that you are taking a hiking trip in Qiaxi Forest Park.*” Subsequently, the distinct written materials validated in the pretest were presented. After the text manipulation, picture stimuli (see Fig. 7) were displayed to participants in the four conditions. The two pictures were identical in landscape composition, perspective, and brightness, with the only difference being with artificial facilities.

This questionnaire comprised three sections. The first section assessed pro-environmental behavior, with artificial facility intrusion serving as the key predictor variable. The measurement items for artificial facility intrusion (α = 0.865) and pro-environmental behavior (α = 0.869) were consistent with those used in Study 1. The second section evaluated aesthetic experience (α = 0.901), place attachment (α = 0.912), and ecological environment value. The items measuring aesthetic experience and place attachment were consistent with those in Study 2, whereas ecological environment value was assessed using a scale adapted from Stern et al.^67^, consisting of 4 items (α = 0.869). Example items included “*We should protect our homeland and the Earth’s biodiversity*,” “*We should reduce pollution and use natural resources rationally*,” “*Protecting natural resources and the environment is beneficial to social development*,” and “*Although humans have the ability to transform nature, they still need to abide by the laws of nature*.” All items were rated on a 7-point Likert scale (1 = strongly disagree, 7 = strongly agree). The third section collected participants’ demographic information (e.g., gender, age).

#### Findings

We performed CFA using AMOS 27.0 The hypothesized three-factor model (Aesthetic Experience, Place Attachment, Pro-environmental Behavior) demonstrated a good fit to the data (χ^2^/df = 1.854, CFI = 0.924, IFI = 0.923, GFI = 0.912, RMSEA = 0.058). All indices meet the recommended thresholds, confirming the structural validity of the scale. Subsequently, this study further evaluated the reliability and convergent validity of the model. The composite reliability (CR) values of each construct ranged from 0.918 to 0.957, significantly exceeding the recommended threshold of 0.70. This indicates that all latent variables have excellent internal consistency. At the same time, the calculation results of AVE showed that the AVE values of “aesthetic experience” (AVE = 0.754), “place attachment” (AVE = 0.824), and “pro-environmental behavior” (AVE = 0.838) were all higher than the judgment standard of 0.50. This confirmed that the latent variables have a significantly stronger explanatory power for their own measurement items than for the measurement error, and the scale has good convergent validity.

***Manipulation checks.*** Two-way ANOVA was conducted to verify the manipulation. For artificial facility intrusion, the degree of intrusion in the non‑intrusion group was significantly lower than that in the intrusion group (M_non-intrusion_= 2.16, SD = 0.953; M_intrusion_ = 4.87, SD = 1.094; F(1, 369) = 648.431, *p* < 0.001, η^2^ = 0.637).There was no significant difference between the low and high ecological environment value groups (M_low_ = 3.42, SD = 1.720; M_high_ = 3.63, SD = 1.675; F(1, 369) = 2.855, *p* = 0.092, η^2^ = 0.008).The interaction effect between the two factors was not significant (*p* = 0.873, η^2^ < 0.001). For ecological environment value, scores in the high group were significantly higher than those in the low group (M_low_ = 3.08, SD = 1.105; M_high_ = 5.41, SD = 0.723; F(1, 369) = 587.270, *p* < 0.001, η^2^ = 0.614).There was no significant difference between the non‑intrusion and intrusion groups (M_non-intrusion_ = 4.34, SD = 1.590; M_intrusion_ = 4.16, SD = 1.391; F(1, 369) = 3.842, *p* = 0.051, η^2^ = 0.010).The interaction effect between the two factors was not significant (*p* = 0.912, η^2^ < 0.001). These results confirmed that experimental manipulations were successful.

***Moderating effect.*** Two-way ANOVA results demonstrated significant main effects of artificial facility intrusion (non‑intrusion vs. intrusion) on all key variables. For pro-environmental behavior, the non‑intrusion group exhibited significantly higher scores than the intrusion group (M_non-intrusion_ = 4.69, SD = 1.362; M_intrusion_ = 3.20, SD = 1.271; F(1, 369) = 119.264, *p* < 0.001, η^2^ = 0.243). For aesthetic experience, the non‑intrusion group scored significantly higher than the intrusion group (M_non-intrusion_ = 4.95, SD = 1.407; M_intrusion_ = 3.85, SD = 1.112; F(1, 369) = 70.771, *p* < 0.001, η^2^ = 0.161). For place attachment, the non‑intrusion group also showed significantly higher scores than the intrusion group (M_non-intrusion_ = 4.87, SD = 1.484; M_intrusion_ = 3.40, SD = 1.312; F(1, 369) = 102.621, *p* < 0.001, η^2^ = 0.217).

**Fig. 8:**
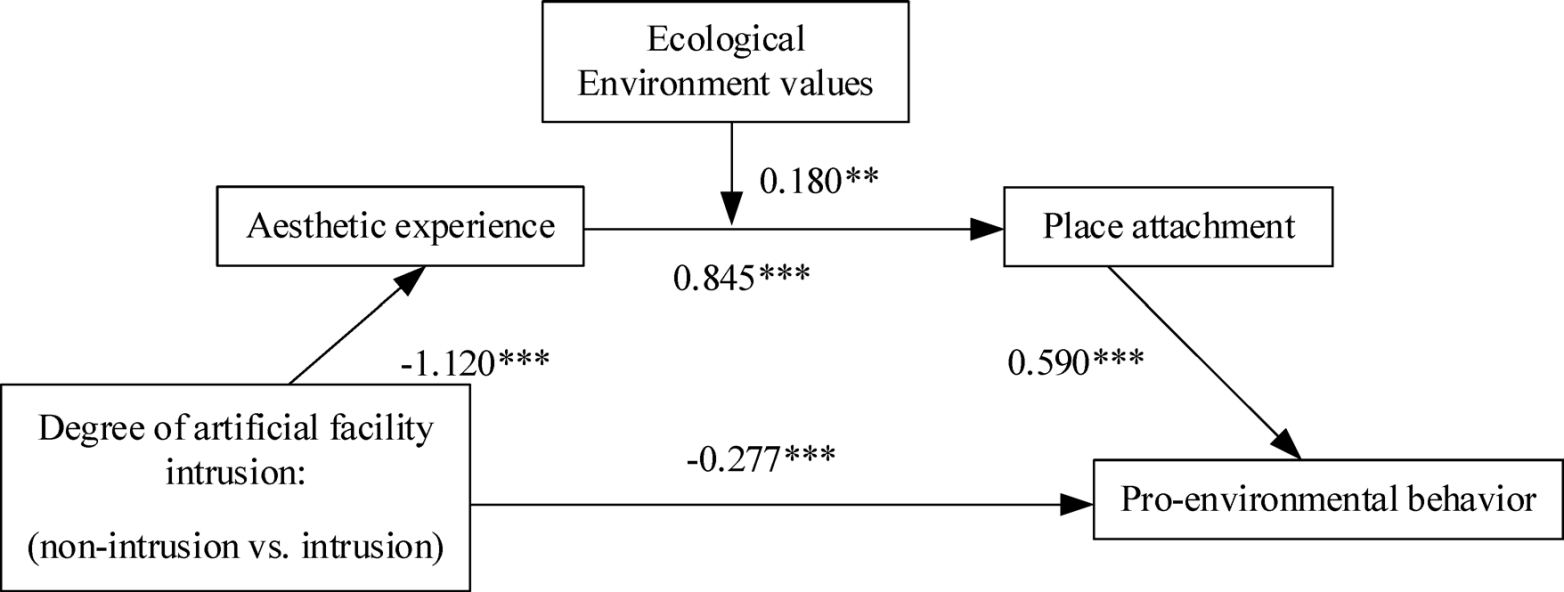
Results of experiment 3.

***Moderated mediation analysis.*** A moderated mediation analysis was conducted using the Bootstrap method (PROCESS, Model 91, n = 5000) to test the hypothesized model. The model of this study is a typical moderated chain mediation model, which conforms to the framework set by Model 91. The mediation effects and detailed results were presented in Table 3 and Fig. 8. For pro-environmental behavior, the moderated mediation effect was significant (moderated mediation index = − 0.119, LLCI = − 0.198, ULCI = − 0.054, 95% CI does not include zero). After controlling for the mediating variables (aesthetic experience and place attachment), the direct negative effect of artificial facility intrusion on pro-environmental behavior remained significant (β = − 0.277, LLCI = − 0.410, ULCI = − 0.145, 95% CI does not include zero).

**Table 3:** Moderating effect test results.

Effect type	Effect	SE	t	Sig	95 per cent CI	
					LLCI	ULCI
Direct effect	− 0.277	0.068	− 4.109	< 0.001	− 0.410	− 0.145
Indirect effect						
IV → AE → PB	− 0.355	0.117			− 0.626	− 0.162
IV → PA → PB	− 0.242	0.081			− 0.416	− 0.103
IV → AE → PA → PB (low values)	− 0.559	0.094			− 0.746	− 0.377
IV → AE → PA → PB (high values)	− 0.678	0.111			− 0.897	− 0.459

IV is the independent variable degree of artificial facility intrusion(non-intrusion vs. intrusion), AE is aesthetic experience, PA is place attachment, PB is pro-environmental behavior.

***Path analysis.*** Path analysis revealed that artificial facility intrusion negatively influenced aesthetic experience (β = − 1.120, LLCI = − 1.379, ULCI = − 0.862, 95% CI does not include zero). Aesthetic experience, in turn, positively influenced place attachment (β = 0.845, LLCI = 0.770, ULCI = 0.921, 95% CI does not include zero), and place attachment further positively influenced pro-environmental behavior (β = 0.590, LLCI = 0.512, ULCI = 0.669, 95% CI does not include zero). Furthermore, ecological environment value negatively influenced place attachment (β = − 0.546, LLCI = − 1.061, ULCI = − 0.031, 95% CI does not include zero). More importantly, the interaction effect between aesthetic experience and ecological environment value on place attachment was significant (β = 0.180, LLCI = 0.068, ULCI = 0.292, 95% CI does not include zero). Specifically, for the low ecological environment value group, the chain mediating effect of aesthetic experience and place attachment was significant (β = − 0.559, LLCI = − 0.746, ULCI = − 0.377, 95% CI does not include zero). For the high ecological environment value group, this chain mediating effect was also significant (β = − 0.678, LLCI = − 0.897, ULCI = − 0.459, 95% CI does not include zero), with the degree of the effect further amplified. These results indicated that as the level of ecological environment value increases, the chain mediating effect of aesthetic experience and place attachment were strengthened. Consequently, Hypotheses H1 through H4 were supported, and Hypothesis H5 was validated.

#### Discussion

Study 3 further investigated the positive moderating role of individuals’ ecological environment value between aesthetic experience and place attachment. For individuals with higher ecological environment value, their aesthetic experience often extends beyond superficial landscape preferences and is closely integrated with profound ecological ethics and sustainability concerns. Accordingly, their aesthetic experience is more likely to generate a strong sense of responsibility, belonging, and protection motivation, thereby significantly enhancing the strength and stability of place attachment. In contrast, for individuals with lower ecological environment value, aesthetic experience tends to be confined to immediate sensory pleasure, and its association with place attachment is relatively weak and susceptible to external influences.

## Conclusions and implications

### Conclusions

This study focuses on the intrinsic relationship between artificial facility intrusion in natural scenic areas and tourists’ pro-environmental behaviors. Through three sequential scenario experiments, it systematically examines the influencing mechanism and boundary conditions of artificial facility intrusion on tourists’ pro-environmental behaviors.

First, artificial facility intrusion has a significant negative impact on tourists’ pro‑environmental behaviors, and this main effect is stable across different types of natural tourism settings. As a heterogeneous intervention in natural landscapes, excessive or inappropriate artificial facilities undermine the integrity and aesthetic harmony of natural scenic areas, creating an environmental signal like a “visual broken window.” Such intrusion not only directly impairs tourists’ aesthetic perception of the landscape but also conveys an implicit signal of lax environmental norms. This, in turn, weakens their environmental concern and sense of responsibility toward the destination, induces environmentally destructive behaviors, and inhibits the emergence of pro‑environmental behaviors.

Second, artificial facility intrusion influences pro-environmental behavior through the chain mediating path of “aesthetic experience—place attachment”. Artificial facility intrusion first disrupts aesthetic harmony and reduces tourists’ sensory pleasure and emotional resonance, thereby weakening their aesthetic experience. High-quality aesthetic experience serves as a key foundation for fostering place attachment. When tourists obtain a harmonious aesthetic experience, they tend to develop affection, attachment, and identification toward the destination. Such attachment leads tourists to regard the destination as a meaningful emotional bond and integrate its environment into their self-concept, thereby stimulating intrinsic motivation to maintain environmental integrity. Place attachment also deepens tourists’ understanding of the ecological characteristics and vulnerability of the destination, enhances their sense of environmental responsibility, and further promotes specific pro-environmental behaviors such as low-carbon travel and waste reduction.

Finally, ecological environment value plays a significant positive moderating role in the relationship between aesthetic experience and place attachment, identifying the key boundary condition of the chain mediating effect. As individuals’ core attitudes toward the natural environment and related issues, ecological environment value endows tourists’ aesthetic experience with richer ecological value connotations.

For individuals with high ecological environment value, their aesthetic experience can be deeply integrated with ecological cognition, thereby being more effectively transformed into place attachment. In contrast, for those with low ecological environment value, their aesthetic experience mostly remains at the perceptual level and is difficult to deepen into place attachment, which reduces the transmission efficiency of the mediating path. Therefore, shaping the public’s ecological value toward the natural environment is crucial for promoting a broader range of pro-environmental behaviors among tourists.

In summary, this study clearly outlines the complete theoretical framework through which artificial facility intrusion influences tourists’ pro-environmental behavior. As a negative environmental stimulus, artificial facility intrusion ultimately reduces pro-environmental behavioral intentions by impairing aesthetic experience and weakening place attachment. Meanwhile, ecological environment value moderates the efficiency with which aesthetic experience is transformed into place attachment. These findings not only address the core issue of whether artificial facility intrusion affects pro-environmental behavior via the “broken window effect”, but also systematically explain the deep relationships among environmental aesthetics, tourist psychology, and pro-environmental behavior, providing empirical evidence for understanding the human-place relationship in tourism.

### Theoretical contributions

Through systematic empirical tests, this study reveals the influencing mechanism and boundary conditions of artificial facility intrusion on tourists’ pro‑environmental behavior and achieves multi‑dimensional theoretical innovations. The specific contributions are as follows:

First, this study expands and deepens the conceptual connotation and empirical scope of “artificial facility intrusion” in tourism environments. Existing literature has mostly focused on the explicit ecological damage and visual conflicts caused by large‑scale, engineered artificial facilities in natural landscapes^16–18^ (Ana del Carmen et al. 2009), while paying insufficient attention to the implicit intrusion effects of routine and scattered artificial facilities within scenic areas. This study clearly proposes that artificial facility intrusion, as an objective environmental cue in the form of a “visual broken window”, harms landscape aesthetic harmony and continuity and inhibits tourists’ pro‑environmental behaviors through internal psychological processes. Meanwhile, this study refines the conceptual definition and research perspective of artificial facility intrusion by introducing the “intrusion” concept from ecology and sociology into tourism research. It explicitly defines artificial facility intrusion as “a mandatory, heterogeneous disturbance to the integrity and harmony of landscapes and tourists’ aesthetic experience”, and reveals its cascading negative effects at the visual, psychological, and behavioral levels, providing a new analytical framework for understanding the interaction between artificial and natural environments in tourism.

Second, this study enriches the theoretical application of the broken window effect in the tourism context and reveals the behavioral transmission mechanism of static environmental disorder. Existing studies on the broken window effect have mostly focused on dynamic and explicit environmental disorder such as littering and graffiti^9^. This study extends it to artificial facility intrusion, a static and systematic environmental disorder, confirming that such implicit “broken windows” can also induce tourists’ non-environmental behavioral tendencies by undermining positive psychological experiences. This extension not only enriches the theoretical connotation and application scenarios of the broken window effect but also provides a new theoretical perspective for understanding the transmission logic of “environmental cues—behavioral norms—individual behavior” in tourism context.

Third, this study shifts the outcome of aesthetic experience from self‑serving behaviors to altruistic behaviors, providing a new research direction for the consequences of aesthetic experience. As an extension of aesthetic research in tourism, the basic concept and measurement dimensions of aesthetic experience have been gradually refined. Previous studies have mostly focused on how aesthetic experience promotes self‑interested outcomes, such as personal satisfaction, loyalty, and revisit intention^23,76^. However, this perspective overlooks the potential of aesthetic experience in stimulating individuals’ altruistic behaviors, especially pro‑environmental behaviors. This study empirically demonstrates that low‑quality aesthetic experience (induced by artificial facility intrusion) can significantly reduce tourists’ place attachment, thereby weakening their pro‑environmental behavioral intentions. These findings highlight the potential power of aesthetic experience in promoting the sustainable development of natural tourism destinations and enrich the theoretical perspectives of sustainable tourism and pro‑environmental behavior research.

Finally, this study constructs and validates an integrated theoretical framework that combines environmental cues, psychological processes, and behavioral responses. Grounded in the broken window theory and the stimulus-organism-response (SOR) model, this study proposes a chain mediating path of “artificial facility intrusion—aesthetic experience—place attachment—pro-environmental behavior” and introduces ecological environment value as a moderating variable. This model not only clarifies the internal mechanism through which negative environmental cues inhibit pro-environmental behavior by undermining aesthetic harmony but also reveals the critical boundary role of individual value orientations. Accordingly, it provides a consistent explanation from external environmental stimuli to individual cognition, affect, and behavior, and promotes the integration and deepening of human-place interaction theory in the tourism context.

### Management insights

First, in the planning and design of artificial facilities, the core principles of minimal intrusion and harmonious integration should be implemented. Artificial facility intrusion that is incompatible with the natural landscape undermines the aesthetic harmony of the landscape, thereby weakening tourists’ pro-environmental intentions. Destination managers should recognize the importance of environmental aesthetics and adopt effective management strategies to prevent the degradation of landscape quality^77^. Therefore, managers must intervene at the source. In the facility planning stage, strict aesthetic and visual impact assessments should be conducted, and design schemes and construction technologies that exert the least disturbance to the integrity of the natural landscape should be prioritized. Facility design should follow the idea that “form follows nature”, striving to coordinate with the surrounding natural environment in terms of material, color, shape, and volume, and avoiding the formation of abrupt visual focal points. For example, trails can be constructed using local stone or eco-friendly wood; the colors of facilities should match the natural landscape; and the design of facilities such as cable cars and viewing platforms should be “hidden in the landscape” rather than “dominating the landscape”. Meanwhile, existing incongruous facilities should be renovated or removed to restore the harmony of the landscape. This practice not only maintains the core aesthetic value of the destination but is also critical to blocking the “visual broken window” effect and conveying positive signals of environmental norms.

Second, efforts should be made to systematically cultivate and enhance tourists’ aesthetic experience, and on this basis, consciously shape their sense of place attachment. Given the chain mediating role of aesthetic experience and place attachment in driving pro-environmental behavior, managers should go beyond merely providing convenient services and strive to create tourism experiences that can trigger deep emotional resonance. On the one hand, the natural and harmonious beauty of the destination can be maximized by optimizing viewing routes, setting up optimal photography spots, and protecting core landscape view corridors. On the other hand, immersive and interactive experience activities should be designed, such as nature education lectures, ecological tours, and place-based cultural events, to guide tourists from passive viewing to active participation and emotional engagement. Providing high-quality, people-oriented services and creating a friendly and tranquil visiting atmosphere also help to solidify tourists’ positive emotions into lasting emotional bonds with the destination. When tourists regard the tourism destination as a cherished place rather than a mere consumption space, their intrinsic motivation to spontaneously protect the local environment will be significantly enhanced.

Finally, emphasis should be placed on actively guiding tourists’ ecological environment values as a lever to enhance management effectiveness. This study finds that individuals’ ecological environment values can significantly strengthen the positive impact of aesthetic experience on place attachment. Therefore, destination management should not be limited to the management of the physical environment but also undertake the responsibility of environmental education. Ecological knowledge, the importance of environmental protection, and the concept of sustainable tourism can be systematically integrated into visitor centers, signage systems, interpretation services, and online platforms. Vivid ecological stories and scientific data on environmental changes can be used to awaken tourists’ ecological awareness and sense of crisis, thereby improving their level of ecological environment values. For tourists with high ecological environment value, managers can provide channels such as voluntary activities and citizen science projects to convert their environmental enthusiasm into specific conservation actions.

### Limitations and future research

First, the main case of this study focuses on a typical destination for nature-based tourism, and it is necessary to verify whether the conclusions can be generalized to cultural tourism destinations. There are substantial differences in landscape characteristics, functional positioning of artificial facilities, and tourists’ experiential needs between different types of tourism destinations, so the influence mechanism of artificial facility intrusion may be heterogeneous. Future research could extend the context to mixed cultural and natural tourism destinations, compare the differential effects of artificial facility intrusion across diverse landscape contexts, and test the universality of the findings. In addition, this study operationalizes artificial facility intrusion as a holistic construct without distinguishing the differential impacts of facility types (e.g., service facilities, commercial facilities, viewing facilities), intrusion forms (visual intrusion, spatial intrusion, functional intrusion), and intrusion intensity. The degree of damage to landscape aesthetic harmony and the psychological impact on tourists may differ significantly across types of artificial facilities. Future research could establish a multi-dimensional measurement system of artificial facility intrusion to precisely identify the unique mechanisms underlying different intrusion dimensions.

Second, the survey data used in this study is collected at a single point in time. Further research is needed to explore whether pro-environmental behavioral intentions change over time and whether such intentions in tourism destinations can spill over to the household context. Future studies may adopt a longitudinal design and collect time-series data to further improve the reliability of the findings and examine the contextual spillover effects of pro-environmental behavior. In addition, this study measures tourists’ pro-environmental behavioral intentions using self-reported assessments. However, the influence of social and moral pressure may lead tourists to overestimate their pro-environmental behavioral intentions, which could further affect the accuracy of the survey data. Therefore, future research could integrate multiple research methods and adopt more objective measurement approaches to assess tourists’ pro-environmental behavioral intentions.

Furthermore, this study also has certain methodological limitations, mainly reflected in the differences between the static image stimuli used in the scenario experiments and real tourism experiences. Although standardized image materials can effectively control extraneous variables and establish causal relationships among variables, the immersive and dynamic experiences obtained by tourists in the field through multi‑sensory channels—including vision, hearing, smell, and touch—are far richer and more complex than can be fully reproduced by static images. These differences may lead to differences in the intensity, depth, and persistence of aesthetic experience induced in this study compared with real situations, thereby affecting the generalizability of the research conclusions to actual tourism settings. To address this limitation, future research could adopt a mixed design combining scenario experiments and field surveys. Causal relationships and mechanisms could be identified in the laboratory using images or virtual reality (VR) techniques, followed by field investigations in real natural scenic areas to collect real‑time experience data from tourists and examine the applicability and boundary conditions of the experimental findings in authentic contexts. Alternatively, 360° panoramic videos, virtual reality, or augmented reality (AR) could be used to provide more immersive and interactive simulated environments that closely approximate real experiences, thereby improving the ecological validity of the experiments. Second, future research could consider incorporating physiological indicators such as eye‑tracking, electroencephalography (EEG), or skin conductance responses to more objectively measure tourists’ attention allocation, emotional arousal, and cognitive load when exposed to different landscapes, providing multi‑dimensional evidence for variable measurement. Meanwhile, qualitative research methods such as in‑depth interviews and focus groups could be applied to explore tourists’ subjective perceptions and deeper psychological mechanisms toward artificial facility intrusion, offering richer qualitative interpretations for the quantitative results and enhancing the accuracy and scientific rigor of the research.

Finally, the limitations of this study are also reflected in the focus of research contexts and applicable populations. The present experiments using image stimuli were conducted in the context of natural tourism destinations and tourist samples, examining the impact of artificial facility intrusion on tourists’ psychology and pro‑environmental behavior in tourism settings. However, this study has not fully considered whether the effect of harmonious natural landscapes as universal aesthetic and environmental cues is confined to the tourism domain, nor has it verified its applicability in non‑tourism contexts (e.g., product packaging in daily life), which constitutes an important limitation of this study. In the tourism context, tourists’ perception of natural landscapes is purposeful and immersive, with the core motivation of experiencing nature and achieving aesthetic satisfaction. In contrast, in daily consumption contexts, consumers’ contact with product packaging is more frequent and fragmented, and their perception logic and emotional engagement with natural elements on packaging may differ significantly from those in tourism contexts. Existing research has not yet revealed the potential impact of such contextual differences on the effects of harmonious natural landscapes.

Accordingly, future research can be further developed from two aspects: context extension and effect verification. First, the research context should be extended from natural tourism destinations to consumption contexts such as product packaging in daily life. Using a similar image-stimulus experimental design, future studies can examine whether the presentation of harmonious natural landscape elements on product packaging—such as natural textures, ecological colors, landscape patterns, and so on—can elicit consumers’ positive aesthetic experience and emotional connection, just as natural landscapes do in tourism contexts, and further influence their decisions including purchase intention and sustainable consumption behavior. Second, attention should be paid to the appropriateness of the presentation forms of harmonious natural landscapes across different contexts. Natural landscapes in tourism contexts are mostly panoramic and immersive images, whereas product packaging is limited by space and function, requiring refined local elements, minimalist patterns, and other condensed forms. Future research should optimize the design of stimulus materials to ensure that the presentation of natural elements is consistent with the practical logic of product packaging while effectively conveying harmonious aesthetic characteristics. Meanwhile, the interference of extraneous variables such as packaging material, product type, and brand awareness should be strictly controlled to improve the reliability of experimental results. In this way, the applicable scope and theoretical depth of research related to harmonious natural landscapes can be enriched, and more universally applicable references can be provided for practical fields including product packaging design and sustainable consumption guidance.

## Data Availability

The datasets used and analyzed during the current study are provided within the supplementary information files. For further data access or correspondence, please refer to the link: https://figshare.com/s/a8abb063155a5cc9112f.
